# Association between dietary supplement use and mortality in cancer survivors with different body mass index and frailty status: a cohort study

**DOI:** 10.3389/fnut.2024.1395362

**Published:** 2024-05-01

**Authors:** Mengqi Zhang, Jia Wang, Xiaoxuan Li, Lihua Zhang, Yixuan Zhang, Zengjin Wen, Jiaqi Zhang, Yuchen Fan, Zhenkang Qiu

**Affiliations:** ^1^Department of Oncology, The Affiliated Hospital of Qingdao University, Qingdao, Shandong, China; ^2^Department of Gastroenterology, The Affiliated Hospital of Qingdao University, Qingdao, Shandong, China; ^3^Department of Medicine, Qingdao University, Qingdao, China; ^4^Interventional Medical Center, The Affiliated Hospital of Qingdao University, Qingdao, Shandong, China

**Keywords:** body mass index, frailty index, dietary supplement, cancer, mortality, National Health and Nutrition Examination Survey

## Abstract

**Background:**

The association between Body Mass Index (BMI), frailty index (FI), and dietary supplement in cancer survivors has been a subject of growing interest. This study investigates the relationship of BMI and FI with mortality in American cancer survivors and explores the impact of dietary supplement usage on different BMI and FI groups.

**Methods:**

Three thousand nine hundred and thirty-two cancer patients from the National Health and Nutrition Examination Survey (NHANES) database were included in the analyses. BMI, FI, and supplement usage were obtained through the NHANES structured survey and the 49-item FI tool. Weighted logistic and Cox proportional hazards models, Kaplan–Meier survival analyses, and propensity score matching (PSM) were used to elucidate the relationships between BMI, FI, dietary supplement, and mortality outcomes.

**Results:**

The study found significant associations between higher BMI and increased frailty (Odds ratio [OR] = 1.04, 95% confidence interval [95% CI], 1.02–1.06). BMI < 25 kg/m^2^ and FI > 0.2 are associated with an increased mortality rate. Dietary supplement use can reduce all-cause and cancer mortality in cancer patients with BMI < 25 kg/m^2^ (Hazard ratio [HR] = 0.63, 95% CI, 0.47–0.84; HR = 0.48, 95% CI, 0.29–0.80) or FI ≤ 0.2 (HR = 0.77, 95% CI, 0.60–0.99; HR = 0.59, 95% CI, 0.39–0.89). In cancer patients with BMI < 25 kg/m^2^ and FI ≤ 0.2, dietary supplement users had lower all-cause and cancer mortality (HR = 0.49, 95% CI, 0.30–0.79; HR = 0.25, 95% CI, 0.10–0.60).

**Conclusion:**

The study revealed a negative correlation between BMI and the FI among the cancer patient cohort as well as their complex impact on mortality and highlighted the role of dietary supplement in cancer prognosis, indicating benefits for non-frail patients with BMI < 25 kg/m^2^.

## 1 Introduction

In 2022, there were over 20.0 million new cancer cases and nearly 9.7 million cancer-related deaths all over the world, making it one of the leading causes of death globally (1). The burden of cancer is substantial and continuously evolving, with prevalence and mortality rates influenced by a myriad of factors including genetic susceptibility, infections, tobacco, alcohol, radiation, dietary habits, lifestyles, and other environmental exposures (2–4). Central to improving cancer outcomes is the identification and understanding of prognostic factors (5).

Body Mass Index (BMI) is widely recognized in clinical settings for its utility in evaluating the general health and nutritional status of patients (6–16). Studies have demonstrated mixed impacts of BMI on cancer patient survival, making it a controversial component in the holistic management of cancer patients (10–16). Beyond BMI, frailty is increasingly recognized in guiding healthcare and predicting clinical outcomes of patients, particularly among the elderly. It represents an individual’s health status and capacity to withstand stressors such as illness or treatment (17). The frailty index (FI) has been identified as a significant predictor of prognosis in many diseases, such as cardiovascular diseases, respiratory illnesses, infections, and cerebrovascular diseases (18–20). Additionally, the role of dietary supplement in the management and prognosis of cancer patients is increasingly being recognized (21–23).

The primary objective of this study is to investigate the associations between BMI, FI, and mortality in a cohort of American cancer patients from the National Health and Nutrition Examination Survey (NHANES) and explore the impact of dietary supplement usage on survival outcomes within different contexts of BMI and FI.

## 2 Materials and methods

### 2.1 Study population and design

This research encompasses the analysis of the NHANES database from 1999 to 2018, which is a comprehensive data collection representing the non-institutionalized, civilian population of the United States through a national, multistage, stratified, clustered probability sampling approach. The National Center for Health Statistics Ethics Review Board sanctioned the survey, and all participants provided their written consent.

**Figure 1:**
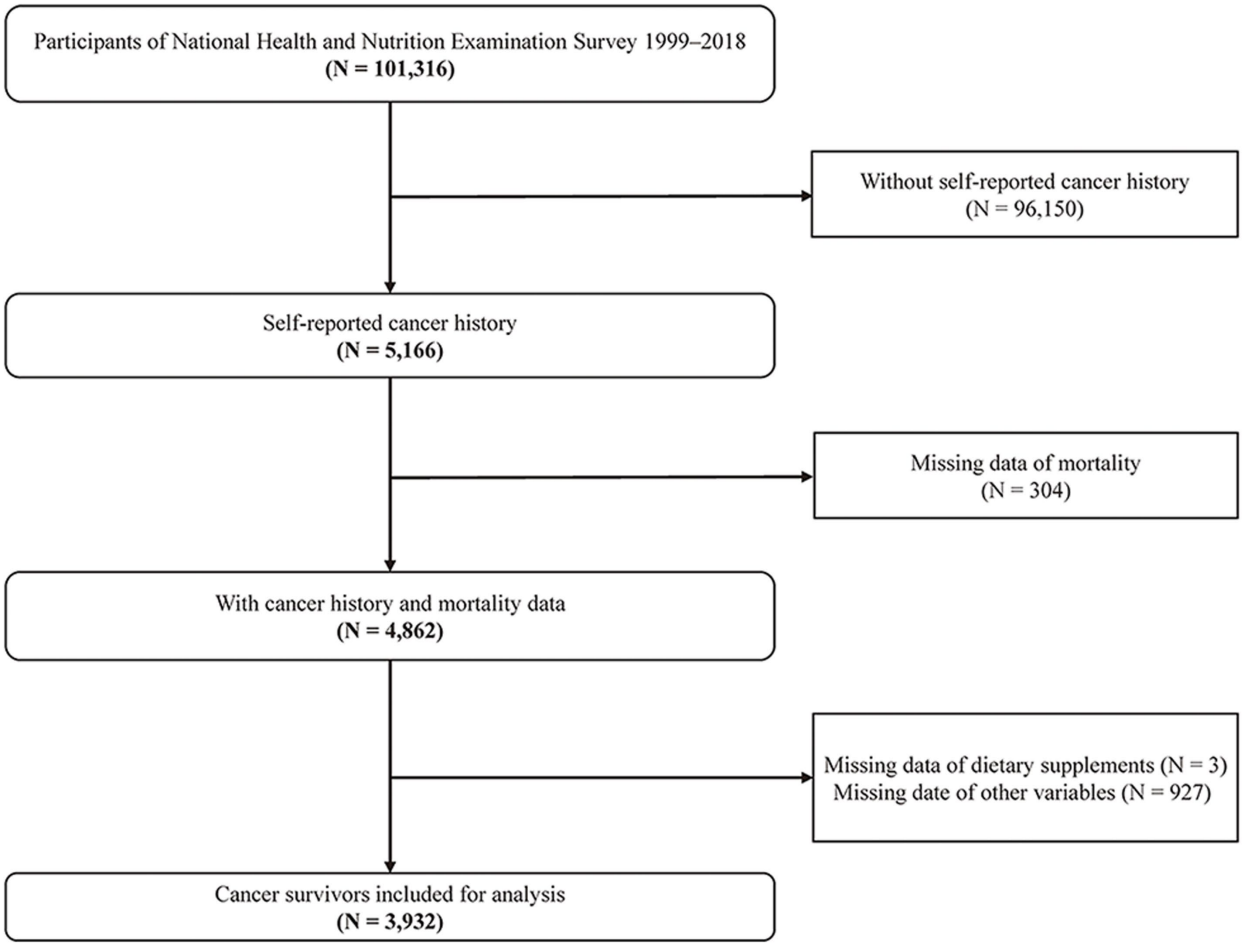
Study flowchart.

The analysis incorporated data from ten NHANES cycles spanning the years 1999 to 2018, initially involving 101,316 individuals. Exclusions were made for 96,150 individuals due to a lack of self-reported cancer history, 304 individuals due to absent follow-up information, three individuals missing data on dietary supplement, and 927 individuals with incomplete data on other variables. Finally, the study included 3,932 participants for the final analysis. The selection process is depicted in Figure 1.

### 2.2 Definition of BMI, FI, and dietary supplement use

BMI was determined by the ratio of weight in kilograms (kg) to the square of height in meters (m^2^). Based on BMI, participants were classified into three categories: normal weight (BMI < 25 kg/m^2^), overweight (25 ≤ BMI < 30 kg/m^2^), and obese (BMI ≥ 30 kg/m^2^). The FI comprised 49 items spanning multiple systems, including cognition, dependence, depression, comorbidities, hospital utilization and general health, physical performance, and anthropometry and laboratory values. The FI score is the ratio of observed deficits to the total possible deficits (24). Details of the FI components and their scoring are available in Supplementary Table S1. Individuals who answered “yes” to the question “Have you used or taken any vitamins, minerals, or other dietary supplement in the past month” in the NHANES survey were categorized as dietary supplement users.

### 2.3 Assessment of mortality

The National Center for Health Statistics (NCHS) provided public use linked mortality files. Mortality status was ascertained by linking the unique study identifier with the National Death Index (last followed up on 31 December 2019, updated in 2022). Causes of death were determined according to the International Statistical Classification of Diseases and Related Health Problems (ICD), tenth revision. This classification system was used to classify cases based on the information on the major cause of death (ICD-10). The main findings of this study were mortality from all-cause, and cancer (codes C00–C97).

### 2.4 Ascertainment of covariates

Detailed information on covariates includes age, gender, race/ethnicity, education level (grades 0–12, high school graduate/GED, some college or above), marital status, smoking, alcohol consumption, Healthy Eating Index-2015 (HEI-2015), physical activity, hypertension, hyperlipidemia and diabetes history, and Charlson Comorbidity Index (CCI). Hypertension was defined as mean systolic blood pressure ≥ 140 mmHg and/or diastolic blood pressure ≥ 90 mmHg, a self-reported diagnosis of hypertension, and/or the use of antihypertensive medication. Hyperlipidemia was defined as triglyceride ≥150 mg/dL, and/or total cholesterol ≥200 mg/dL, and/or low-density lipoprotein ≥130 mg/dL, and/or high-density lipoprotein <40 mg/dL in males or < 50 mg/dL in females, and/or the use of lipid-lowering drug. Diabetes was defined as HbA1c ≥ 6.5%, a self-reported diagnosis of diabetes, and/or the use of anti-diabetic medication. CCI was calculated according to questionnaire survey and examination (25).

### 2.5 Statistical analyses

All statistical analyses were conducted by NHANES analysis and reporting criteria. The variance inflation factor (VIF) was applied to assess multicollinearity, with a VIF value above 10 indicating significant multicollinearity (26). The analysis revealed no substantial multicollinearity within this study (Supplementary Table S2).

The accumulation of person-years started from the date of enrollment until the date of either death or censoring. Restricted cubic splines, incorporating three knots located at the 5th, 50th, and 95th percentiles, were employed to model non-linear relationships within the data. Additionally, a likelihood ratio test was conducted to compare the model that includes both linear and cubic spline terms against a model featuring only a linear term. Three weighted logistics regression models were utilized to explore the association between BMI and FI. Three weighted Cox proportional hazard models were constructed to study the relationships between dietary supplement and mortality. Kaplan–Meier survival analyses were utilized to investigate the survival differences in different BMI and FI groups. To further ensure the robustness of the findings, four sensitivity analyses were conducted. Firstly, a propensity score matching (PSM) analysis at a 1:1 ratio was implemented to equate differences between users and non-users of dietary supplement. To conduct sensitivity analyses to access the stability of the results, individuals who were under 65 years old, over 80 years old, had a BMI less than 18.5 kg/m^2^, or died within 2 years of follow-up were excluded separately. Additionally, the relationship between dietary supplement usage and mortality was reevaluated without considering the complexity of the sampling design.

All statistical assessments were performed using a two-sided approach, with a *p-*value <0.05 denoting statistical significance. The analyses were conducted using R 4.3.1 software.

## 3 Results

### 3.1 Population characteristics

Table 1 presents the study population’s baseline characteristics according to the use of dietary supplement in the past 30 days. The weighted mean age of the study population was 61.77 years (confidence interval [95% CI], 61.40–62.14 years), and 2,016 participants were females (weighted percentage [WP], 54.92%). Dietary supplement users were more likely to be younger, male, non-White, married, and current smokers, and have a lower level of educational attainment, HEI-2015, and physical activity (all *p-*value <0.05). There were 1,101 (WP, 29.29%), 1,410 (WP, 35.55%), and 1,421 (WP, 35.16%) participants in the BMI < 25 kg/m^2^, 25 ≤ BMI < 30 kg/m^2^, and BMI ≥ 30 kg/m^2^, respectively, and 2,145 (WP, 62.75%) and 1,787 (WP, 37.25%) in the non-frail and frail groups, respectively, all of which were not statistically significant between the dietary supplement users and non-users groups.

**Table 1:** Characteristics of US adults according to the use of dietary supplement in the past 30 days, NHANES 1999–2018*.

**Characteristics**	**Overall (*N* = 3,932)**	**Use of dietary supplement (*N* = 2,700)**	**No use of dietary supplement (*N* = 1,232)**	***p-*value**
**Age, years, mean (SE)**	61.77 (0.37)	58.57 (0.59)	62.99 (0.44)	<0.001
**Gender, *n* (%)**				0.005
Female	2,016 (54.92)	589 (50.02)	1,427 (56.79)	
Male	1,916 (45.08)	643 (49.98)	1,273 (43.21)	
**Race/ethnicity, *n* (%)**				<0.001
Non-Hispanic White	2,733 (86.62)	737 (81.11)	1,996 (88.73)	
Non-Hispanic Black	554 (5.44)	241 (8.68)	313 (4.21)	
Mexican	262 (2.31)	121 (3.79)	141 (1.75)	
Other	383 (5.62)	133 (6.42)	250 (5.32)	
**Education, *n* (%)**				<0.001
Grades 0–12	833 (13.02)	367 (17.87)	466 (11.17)	
High school graduate/GED	896 (21.33)	311 (27.30)	585 (19.05)	
Some colleges or above	2,203 (65.64)	554 (54.83)	1,649 (69.78)	
**Marital status, *n* (%)**				0.026
Coupled	1,654 (35.85)	569 (39.22)	1,085 (34.56)	
Single or separated	2,278 (64.15)	663 (60.78)	1,615 (65.44)	
**Smoking** ^**†**^ **, *n* (%)**				<0.001
Current smokers	612 (16.33)	288 (25.58)	324 (12.79)	
Former smokers	1,582 (38.44)	461 (34.53)	1,121 (39.93)	
Non smokers	1,738 (45.23)	483 (39.90)	1,255 (47.27)	
**Alcohol consumption, *n* (%)**				0.742
Yes	888 (26.23)	269 (25.75)	619 (26.42)	
No	3,044 (73.77)	963 (74.25)	2,081 (73.58)	
**BMI, kg/m** ^**2**^ **, *n* (%)**				0.535
BMI < 25 kg/m^2^	1,101 (29.29)	321 (28.65)	780 (29.53)	
25 ≤ BMI < 30 kg/m^2^	1,410 (35.55)	430 (34.24)	980 (36.06)	
BMI ≥ 30 kg/m^2^	1,421 (35.16)	481 (37.11)	940 (34.41)	
**Frailty, *n* (%)**				0.052
Frailty index ≤0.2 (Non-frail)	2,145 (62.75)	616 (59.79)	1,529 (63.89)	
Frailty index >0.2 (Frail)	1,787 (37.25)	616 (40.21)	1,171 (36.11)	
**HEI–2015** ^**‡**^ **, *n* (%)**				< 0.001
<46.01	1,311 (31.77)	511 (41.38)	800 (28.09)	
46.02–58.90	1,310 (34.86)	400 (34.98)	910 (34.81)	
≥58.91	1,311 (33.38)	321 (23.64)	990 (37.10)	
**Physical activity** ^**§**^ **, *n* (%)**				0.040
Yes	1,916 (51.68)	547 (47.94)	1,369 (53.11)	
No	2,016 (48.32)	685 (52.06)	1,331 (46.89)	
**Hypertension history, *n* (%)**				0.050
Yes	2,517 (57.29)	772 (53.33)	1,745 (58.80)	
No	1,415 (42.71)	460 (46.67)	955 (41.20)	
**Hyperlipidemia history, *n* (%)**				0.263
Yes	3,093 (78.61)	949 (77.05)	2,144 (79.21)	
No	839 (21.39)	283 (22.95)	556 (20.79)	
**Diabetes history, *n* (%)**				0.125
Yes	900 (17.57)	320 (19.64)	580 (16.78)	
No	3,032 (82.43)	912 (80.36)	2,120 (83.22)	
**CCI, *n* (%)**				0.198
0	140 (3.98)	34 (2.88)	106 (4.40)	
1	151 (3.61)	32 (3.07)	119 (3.82)	
2	1,163 (35.29)	402 (38.50)	761 (34.07)	
≥ 3	2,478 (57.12)	764 (55.55)	1,714 (57.72)	

^*^Means and percentages were adjusted for survey weights of NHANES. ^†^Smoking was defined as smoking at least 100 cigarettes during their lifetime. ^‡^HEI–2015 was calculated to measure adherence to the 2015 Dietary Guidelines for Americans with a higher score corresponding to a higher–quality diet. ^§^The active participants included those who met the recommended physical activity levels of ≥600 MET minutes/week according to the Center for Disease Control and Prevention (CDC) Physical Activity Guidelines for Americans. NHANES, National Health and Nutrition Examination Survey; SE, standard error; GED, general equivalency diploma; BMI, body mass index; HEI, Healthy Eating Index; CCI, Charlson Comorbidity Index.

### 3.2 Associations between BMI and frailty in cancer patients

**Figure 2:**
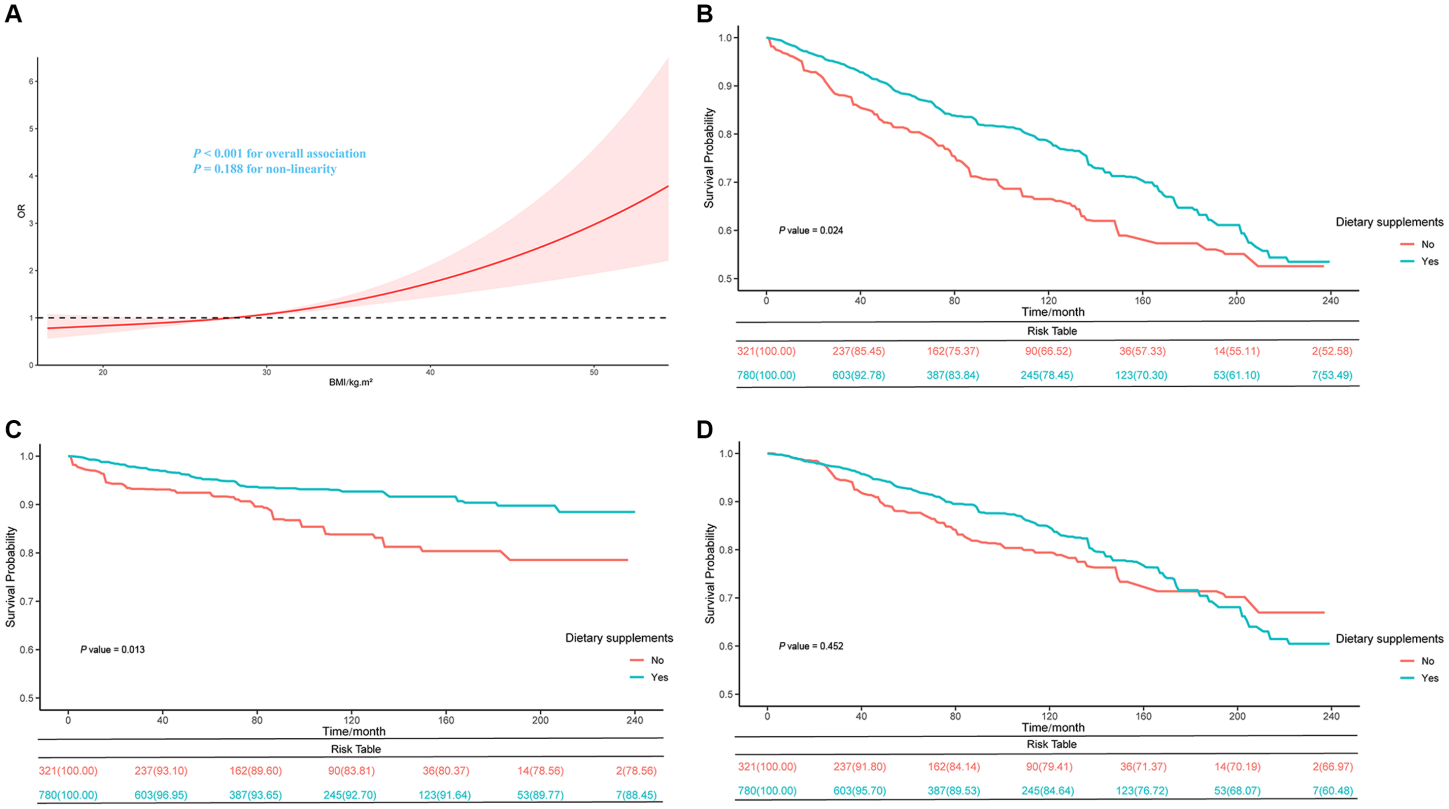
Restricted cubic spline curve of the relationships between BMI and FI in cancer patients **(A)** and Kaplan–Meier survival curves on all-cause **(B)**, cancer **(C)**, and non-cancer **(D)** mortality between dietary supplement users and non-users in BMI < 25 kg/m^2^ group.

Table 2 displays the associations between BMI and FI by the survey-weighted logistics regression models. The univariate and multivariate analyses adjusted for confounding factors indicate a significantly higher frailty risk in groups with 25 ≤ BMI < 30 kg/m^2^ and BMI ≥ 30 kg/m^2^ compared to those with BMI < 25 kg/m^2^ (*p-*value <0.05). In the fully adjusted model (model 2), compared to the normal weight group, the overweight (Odds ratio [OR] = 1.28; 95% CI, 1.01–1.64) and obese group (OR = 1.61; 95% CI, 1.24–2.09, *p* for trend <0.001) were associated with higher FI. The multivariate-adjusted ORs for every 1 kg/m^2^ in BMI in the association with FI was 1.04 (95% CI, 1.02–1.06). Furthermore, restricted cubic spline curves further visualize the relationships between BMI and FI (Figure 2A). After adjusting for confounders, a significant positive linear relationship was observed between BMI and FI (*p* value for overall <0.001, *p* value for non-linearity = 0.188).

**Table 2:** Survey-weighted associations between BMI (kg/m^2^) and frailty index.

	Univariable model		Model 1		Model 2	
	*OR* (*95% CI*)	***p-***value	*OR* (*95% CI*)	***p-***value	*OR* (*95% CI*)	***p-***value
BMI < 25 kg/m^2^	1 [Reference]	/	1 [Reference]	/	1 [Reference]	/
25 ≤ BMI < 30 kg/m^2^	1.45 (1.16,1.82)	0.001	1.48 (1.17,1.87)	0.001	1.28 (1.01,1.64)	0.045
BMI ≥ 30 kg/m^2^	2.22 (1.78,2.77)	<0.001	2.42 (1.92,3.06)	<0.001	1.61 (1.24,2.09)	<0.001
*P* for trend	/	<0.001	/	<0.001	/	<0.001
Per 1 kg/m^2^ increase	1.06 (1.05,1.08)	< 0.001	1.07 (1.05,1.09)	< 0.001	1.04 (1.02,1.06)	< 0.001

Model 1 was adjusted for age, gender, race/ethnicity, education level, and marital status; Model 2 was additionally adjusted for smoking, alcohol consumption, HEI-2015, physical activity, BMI, hypertension, hyperlipidemia and diabetes history, and CCI. CVD, cardiovascular disease; BMI, body mass index; OR, odds ratio; CI, confidence interval; HEI, Healthy Eating Index, CCI, Charlson Comorbidity Index.

### 3.3 Associations of BMI and FI with mortality in cancer patients

The research observed significant associations between BMI, FI, and mortality in cancer patients (Table 3). Patients in the 25 ≤ BMI < 30 kg/m^2^ group exhibited a reduced risk of all-cause mortality (Hazard ratio [HR] = 0.78, 95% CI, 0.65–0.94, *p-*value = 0.008) and cancer-specific mortality (HR = 0.73, 95% CI, 0.58–0.92, *p-*value = 0.007) compared to the reference group (BMI < 25 kg/m^2^). Notably, patients with a BMI ≥ 30 kg/m^2^ also showed a lower risk of all-cause (HR = 0.71, 95% CI, 0.55–0.91, *p-*value = 0.006) and cancer mortality (HR = 0.67, 95% CI, 0.50–0.89, *p-*value = 0.005), although the association with non-cancer mortality was not statistically significant (HR = 0.81, 95% CI, 0.56–1.19, *p-*value = 0.285). Regarding the FI, patients with a FI > 0.2 were associated with substantially increased risks of all-cause (HR = 2.22, 95% CI, 1.86–2.66, *p-*value<0.001), cancer (HR = 2.46, 95% CI, 1.93–3.13, *p-*value<0.001), and non-cancer mortality (HR = 1.81, 95% CI, 1.42–2.31, *p-*value<0.001) compared to those with a FI ≤ 0.2.

**Table 3:** Survey-weighted associations of BMI (kg/m^2^) and frailty index with mortality.

	All-cause mortality		Cancer mortality		Non-cancer mortality	
	*HR (95% CI)*	***p-***value	*HR (95% CI)*	***p-***value	*HR (95% CI)*	***p-***value
**BMI**						
BMI < 25 kg/m^2^	1 [Reference]	/	1 [Reference]	/	1 [Reference]	/
25 ≤ BMI < 30 kg/m^2^	0.78 (0.65,0.94)	0.008	0.73 (0.58,0.92)	0.007	0.90 (0.64,1.27)	0.553
BMI ≥ 30 kg/m^2^	0.71 (0.55,0.91)	0.006	0.67 (0.50,0.89)	0.005	0.81 (0.56,1.19)	0.285
**Frailty index**						
Frailty index ≤0.2	1 [Reference]	/	1 [Reference]	/	1 [Reference]	/
Frailty index >0.2	2.22 (1.86,2.66)	<0.001	2.46 (1.93,3.13)	<0.001	1.81 (1.42,2.31)	<0.001

Adjusted for age, gender, race/ethnicity, education level, marital status, smoking, alcohol consumption, HEI-2015, physical activity, BMI, hypertension, hyperlipidemia and diabetes history, and CCI. CVD, cardiovascular disease; BMI, body mass index; HR, hazard ratio; CI, confidence interval; HEI, Healthy Eating Index, CCI, Charlson Comorbidity Index.

### 3.4 Associations of dietary supplement with mortality and survival in different BMI or frailty groups

During a median of 7.63 years of follow-up, 1,211 deaths (WP, 23.96%; 95% CI, 21.78–26.14%) were documented. Among these, there were 795 deaths attributed to cancer events (WP of 8.05%; 95% CI, 7.01–9.08%).

After adjusting for all covariates, in the BMI < 25 kg/m^2^ group, dietary supplement users had lower risks of all-cause (HR = 0.63; 95% CI, 0.47–0.84) and cancer mortality (HR = 0.48; 95% CI, 0.29–0.80) compared to dietary supplement non-users. However, we found no significant association between dietary supplement use and lower mortality in the overweight and obese group (Supplementary Table S3). Moreover, in the group with BMI < 25 kg/m^2^, the Kaplan–Meier survival curves related to all-cause mortality and cancer mortality showed that dietary supplement users had a higher survival rate compared to non-users of dietary supplement (Figures 2B,C). No difference in non-cancer mortality was observed between the two groups(Figure 2D). In the non-frail group, dietary supplement users had lower risks of all-cause (HR = 0.77; 95% CI, 0.60–0.90) and cancer mortality (HR = 0.59; 95% CI, 0.39–0.89) compared to dietary supplement non-users. No significant difference in mortality was observed within the frail group (Supplementary Table S4). Additionally, by combining the three BMI groups with the two frailty groups into six combined categories, it was observed that within the BMI < 25 kg/m^2^ and non-frail group, dietary supplement users had a lower risk of all-cause mortality (HR = 0.49; 95% CI, 0.30–0.79) and cancer mortality (HR = 0.25; 95% CI, 0.10–0.60) compared to non-users. No significant association between dietary supplement use and lower mortality rates was found in the other five groups (Table 4).

**Table 4:** Survey-weighted associations between dietary supplement and all-cause, cancer, and non-cancer mortality among cancer survivors in different BMI (kg/m^2^) and frailty groups.

	Death, *n*	Weighted death (%)	Univariable model		Model 1		Model 2	
			*HR* (*95% CI*)	***p-***value	*HR* (*95% CI*)	***p-***value	*HR* (*95% CI*)	***p-***value
**BMI < 25** kg/m^2^**and frailty index ≤ 0.2 (Non-frail)**								
**All-cause mortality**								
No	61	22.84	1 [Reference]	/	1 [Reference]	/	1 [Reference]	/
Yes	129	17.24	0.70 (0.45,1.09)	0.118	0.62 (0.40,0.95)	0.030	0.49 (0.30,0.79)	0.004
**Cancer mortality**								
No	21	9.38	1 [Reference]	/	1 [Reference]	/	1 [Reference]	/
Yes	34	4.44	0.46 (0.24,0.90)	0.023	0.40 (0.20,0.82)	0.013	0.25 (0.10,0.60)	0.002
**Non-cancer mortality**								
No	40	13.46	1 [Reference]	/	1 [Reference]	/	1 [Reference]	/
Yes	95	12.80	0.87 (0.48,1.56)	0.631	0.81 (0.44, 1.48)	0.487	0.78 (0.39,1.53)	0.467
**BMI < 25** kg/m^2^**and frailty index > 0.2 (Frail)**								
**All-cause mortality**								
No	72	50.62	1 [Reference]	/	1 [Reference]	/	1 [Reference]	/
Yes	145	47.40	0.84 (0.57,1.23)	0.372	0.75 (0.53,1.07)	0.110	0.83 (0.57,1.20)	0.326
**Cancer mortality**								
No	33	21.54	1 [Reference]	/	1 [Reference]	/	1 [Reference]	/
Yes	45	13.66	0.59 (0.29,1.20)	0.142	0.60 (0.30,1.18)	0.137	0.66 (0.33,1.33)	0.244
**Non-cancer mortality**								
No	39	29.07	1 [Reference]	/	1 [Reference]	/	1 [Reference]	/
Yes	100	33.74	1.03 (0.65,1.63)	0.901	0.83 (0.53,1.30)	0.408	0.91 (0.55,1.51)	0.726
**25 ≤ BMI < 30** kg/m^2^**and frailty index ≤ 0.2 (Non-frail)**								
**All-cause mortality**								
No	54	18.00	1 [Reference]	/	1 [Reference]	/	1 [Reference]	/
Yes	138	20.19	1.03 (0.70,1.51)	0.896	0.90 (0.65,1.26)	0.549	1.07 (0.73,1.57)	0.715
**Cancer mortality**								
No	26	8.71	1 [Reference]	/	1 [Reference]	/	1 [Reference]	/
Yes	40	7.71	0.83 (0.44,1.55)	0.559	0.73 (0.39,1.36)	0.319	0.79 (0.41, 1.51)	0.471
**Non-cancer mortality**								
No	28	9.29	1 [Reference]	/	1 [Reference]	/	1 [Reference]	/
Yes	98	12.47	1.21 (0.67,2.18)	0.1529	1.10 (0.65,1.87)	0.713	1.38 (0.73,2.60)	0.317
**25 ≤ BMI < 30** kg/m^2^**and frailty index > 0.2 (Frail)**								
**All-cause mortality**								
No	91	40.79	1 [Reference]	/	1 [Reference]	/	1 [Reference]	/
Yes	175	35.42	1.05 (0.71,1.55)	0.812	1.00 (0.68,1.47)	0.991	1.07 (0.72,1.59)	0.733
**Cancer mortality**								
No	36	15.17	1 [Reference]	/	1 [Reference]	/	1 [Reference]	/
Yes	51	8.79	0.68 (0.39,1.20)	0.184	0.69 (0.46,1.04)	0.212	0.72 (0.39,1.32)	0.287
**Non-cancer mortality**								
No	55	25.62	1 [Reference]	/	1 [Reference]	/	1 [Reference]	/
Yes	124	26.63	1.27 (0.80,2.00)	0.312	1.13 (0.74,1.73)	0.567	1.22 (0.79,1.89)	0.376
**BMI ≥ 30** kg/m^2^**and frailty index ≤ 0.2 (Non-frail)**								
**All-cause mortality**								
No	36	12.19	1 [Reference]	/	1 [Reference]	/	1 [Reference]	/
Yes	65	9.80	0.86 (0.52,1.43)	0.559	0.77 (0.46,1.28)	0.310	0.70 (0.41,1.21)	0.201
**Cancer mortality**								
No	19	5.18	1 [Reference]	/	1 [Reference]	/	1 [Reference]	/
Yes	28	4.02	0.81 (0.41,1.60)	0.552	0.65 (0.33,1.30)	0.223	0.57 (0.28,1.13)	0.107
**Non-cancer mortality**								
No	17	7.01	1 [Reference]	/	1 [Reference]	/	1 [Reference]	/
Yes	37	5.77	0.89 (0.43,1.85)	0.763	0.86 (0.43,1.74)	0.684	0.84 (0.40,1.78)	0.645
**BMI ≥ 30** kg/m^2^**and frailty index > 0.2 (Frail)**								
**All-cause mortality**								
No	94	29.37	1 [Reference]	/	1 [Reference]	/	1 [Reference]	/
Yes	151	30.69	1.19 (0.76,1.84)	0.448	1.00 (0.61,1.64)	0.995	1.00 (0.65,1.56)	0.987
**Cancer mortality**								
No	33	10.58	1 [Reference]	/	1 [Reference]	/	1 [Reference]	/
Yes	59	9.05	0.94 (0.55,1.62)	0.824	0.92 (0.53,1.60)	0.764	1.00 (0.57,1.74)	0.996
**Non-cancer mortality**								
No	61	18.79	1 [Reference]	/	1 [Reference]	/	1 [Reference]	/
Yes	101	20.64	1.33 (0.80,2.19)	0.268	1.05 (0.56,1.97)	0.888	1.01 (0.58,1.75)	0.984

Model 1 was adjusted for age, gender, race/ethnicity, education level, and marital status; Model 2 was additionally adjusted for smoking, alcohol consumption, HEI-2015, physical activity, BMI, hypertension, hyperlipidemia and diabetes history, and CCI. CVD, cardiovascular disease; BMI, body mass index; HR, hazard ratio; CI, confidence interval; HEI, Healthy Eating Index; CCI, Charlson Comorbidity Index.

### 3.5 Sensitivity analyses

The results remained robust after PSM analysis (Supplementary Table S5), excluding participants less than 65 years old (Table 5), excluding participants with BMI < 18.5 kg/m^2^ (Table 6), excluding deaths with a follow-up period of fewer than 2 years (Supplementary Table S6), excluding participants over 80 years old (Supplementary Table S7), and repeating the main analyses without consideration of complex sampling designs (Supplementary Table S8).

**Table 5:** Sensitivity analysis of the associations between dietary supplements and all-cause, cancer and non-cancer mortality among cancer survivors in different BMI (kg/m^2^) or frailty groups after exclusion of participants less than 65 years old.

	Death, *n*	Weighted death (%)	Univariable model		Model 1		Model 2	
			*HR* (*95%CI*)	*p-*value	*HR* (*95%CI*)	*p-*value	*HR* (*95%CI*)	*p-*value
**BMI < 25 kg/m** ^**2**^								
**All-cause mortality**								
No	101	55.11	1 [Reference]	/	1 [Reference]	/	1 [Reference]	/
Yes	237	38.95	0.65 (0.46,0.92)	0.014	0.69 (0.49,0.97)	0.032	0.69 (0.50,0.94)	0.018
**Cancer mortality**								
No	38	19.61	1 [Reference]	/	1 [Reference]	/	1 [Reference]	/
Yes	62	9.70	0.46 (0.28,0.76)	0.003	0.54 (0.32,0.93)	0.025	0.51 (0.29,0.87)	0.014
**Non-cancer mortality**								
No	63	35.50	1 [Reference]	/	1 [Reference]	/	1 [Reference]	/
Yes	175	29.26	0.76 (0.49,1.18)	0.219	0.76 (0.49,1.19)	0.232	0.80 (0.53,1.22)	0.303
**25 ≤ BMI < 30 kg/m** ^**2**^								
**All-cause mortality**								
No	114	46.36	1 [Reference]	/	1 [Reference]	/	1 [Reference]	/
Yes	278	38.27	0.97 (0.72,1.30)	0.829	1.00 (0.77,1.29)	0.991	1.15 (0.86,1.52)	0.343
**Cancer mortality**								
No	44	17.26	1 [Reference]	/	1 [Reference]	/	1 [Reference]	/
Yes	71	10.25	0.69 (0.42,1.11)	0.127	0.76 (0.45,1.26)	0.282	0.84 (0.50,1.43)	0.527
**Non-cancer mortality**								
No	70	29.10	1 [Reference]	/	1 [Reference]	/	1 [Reference]	/
Yes	207	28.02	1.14 (0.80,1.61)	0.470	1.13 (0.80,1.58)	0.486	1.29 (0.90,1.86)	0.168
**BMI ≥ 30 kg/m** ^**2**^								
**All-cause mortality**								
No	98	37.26	1 [Reference]	/	1 [Reference]	/	1 [Reference]	/
Yes	172	28.82	0.79 (0.54,1.15)	0.217	0.87 (0.60,1.24)	0.436	0.85 (0.62,1.15)	0.293
**Cancer mortality**								
No	38	15.41	1 [Reference]	/	1 [Reference]	/	1 [Reference]	/
Yes	54	8.78	0.58 (0.34,0.98)	0.041	0.62 (0.36,1.06)	0.078	0.58 (0.34,0.97)	0.039
**Non-cancer mortality**								
No	60	21.85	1 [Reference]	/	1 [Reference]	/	1 [Reference]	/
Yes	118	20.04	0.94 (0.61,1.45)	0.778	1.05 (0.69,1.59)	0.821	1.04 (0.70,1.56)	0.833
**Frailty index ≤ 0.2 (Non-frail)**								
**All-cause mortality**								
No	117	35.90	1 [Reference]	/	1 [Reference]	/	1 [Reference]	/
Yes	294	26.64	0.73 (0.55,0.98)	0.034	0.82 (0.61,1.10)	0.194	0.81 (0.62,1.06)	0.126
**Cancer mortality**								
No	50	15.14	1 [Reference]	/	1 [Reference]	/	1 [Reference]	/
Yes	82	8.13	0.53 (0.34,0.83)	0.005	0.61 (0.39,0.96)	0.032	0.60 (0.39,0.94)	0.026
**Non-cancer mortality**								
No	67	20.76	1 [Reference]	/	1 [Reference]	/	1 [Reference]	/
Yes	212	18.51	0.88 (0.60,1.28)	0.495	0.97 (0.66,1.44)	0.898	1.00 (0.67,1.50)	0.995
**Frailty index > 0.2 (Frail)**								
**All-cause mortality**								
No	196	56.65	1 [Reference]	/	1 [Reference]	/	1 [Reference]	/
Yes	393	48.16	1.02 (0.79,1.33)	0.861	0.94 (0.74,1.20)	0.624	0.98 (0.77,1.24)	0.845
**Cancer mortality**								
No	70	19.68	1 [Reference]	/	1 [Reference]	/	1 [Reference]	/
Yes	105	11.76	0.68 (0.45,1.04)	0.078	0.72 (0.46,1.14)	0.162	0.75 (0.48,1.17)	0.203
**Non-cancer mortality**								
No	126	36.97	1 [Reference]	/	1 [Reference]	/	1 [Reference]	/
Yes	288	36.40	1.21 (0.90,1.61)	0.200	1.04 (0.79,1.38)	0.756	1.09 (0.82,1.44)	0.557

Model 1 was adjusted for age, gender, race/ethnicity, education level, and marital status; Model 2 was additionally adjusted for smoking, alcohol consumption, HEI-2015, physical activity, hypertension, hyperlipidemia and diabetes history, CCI, and BMI or frailty index (if not already stratified). CVD, cardiovascular disease; BMI, body mass index; HR, hazard ratio; CI, confidence interval; HEI, Healthy Eating Index; CCI, Charlson Comorbidity Index.

**Table 6:** Sensitivity analysis of the associations between dietary supplements and all-cause, cancer and non-cancer mortality among cancer survivors in different BMI (kg/m^2^) or frailty groups after exclusion of participants with BMI < 18.5 kg/m^2^.

	Death, *n*	Weighted death (%)	Univariable model		Model 1		Model 2	
			*HR* (*95%CI*)	*p-*value	*HR* (*95%CI*)	*p-*value	*HR* (*95%CI*)	*p-*value
**18.5 < BMI < 25 kg/m** ^**2**^								
**All-cause mortality**								
No	123	31.76	1 [Reference]	/	1 [Reference]	/	1 [Reference]	/
Yes	255	24.14	0.69 (0.50,0.95)	0.024	0.61 (0.45,0.83)	0.001	0.63 (0.47,0.85)	0.003
**Cancer mortality**								
No	50	13.75	1 [Reference]	/	1 [Reference]	/	1 [Reference]	/
Yes	72	6.43	0.44 (0.27,0.73)	0.001	0.45 (0.27,0.76)	0.003	0.44 (0.26,0.75)	0.003
**Non-cancer mortality**								
No	73	18.01	1 [Reference]	/	1 [Reference]	/	1 [Reference]	/
Yes	183	17.11	0.88 (0.58,1.35)	0.568	0.72 (0.48,1.09)	0.122	0.80 (0.53,1.19)	0.269
**25 ≤ BMI < 30 kg/m** ^**2**^								
**All-cause mortality**								
No	145	25.76	1 [Reference]	/	1 [Reference]	/	1 [Reference]	/
Yes	313	25.77	1.01 (0.79,1.30)	0.911	0.95 (0.75,1.21)	0.704	1.13 (0.87,1.45)	0.363
**Cancer mortality**								
No	62	10.91	1 [Reference]	/	1 [Reference]	/	1 [Reference]	/
Yes	91	8.11	0.76 (0.53,1.10)	0.151	0.75 (0.51,1.11)	0.148	0.85 (0.57,1.27)	0.433
**Non-cancer mortality**								
No	83	14.85	1 [Reference]	/	1 [Reference]	/	1 [Reference]	/
Yes	222	17.67	1.20 (0.84,1.71)	0.317	1.10 (0.78,1.56)	0.582	1.30 (0.89,1.89)	0.168
**BMI ≥ 30 kg/m** ^**2**^								
**All-cause mortality**								
No	130	20.75	1 [Reference]	/	1 [Reference]	/	1 [Reference]	/
Yes	216	19.15	0.98 (0.70,1.38)	0.925	0.90 (0.62,1.29)	0.552	0.91 (0.64,1.28)	0.581
**Cancer mortality**								
No	52	7.87	1 [Reference]	/	1 [Reference]	/	1 [Reference]	/
Yes	78	6.27	0.85 (0.52,1.37)	0.498	0.80 (0.49,1.32)	0.382	0.89 (0.56,1.42)	0.623
**Non-cancer mortality**								
No	78	12.88	1 [Reference]	/	1 [Reference]	/	1 [Reference]	/
Yes	138	12.88	1.07 (0.73,1.57)	0.741	0.95 (0.61,1.50)	0.838	0.90 (0.57,1.42)	0.658
**Frailty index ≤ 0.2 (Non-frail)**								
**All-cause mortality**								
No	147	17.39	1 [Reference]	/	1 [Reference]	/	1 [Reference]	/
Yes	326	16.09	0.89 (0.68,1.17)	0.398	0.80 (0.62,1.03)	0.078	0.80 (0.63,1.02)	0.072
**Cancer mortality**								
No	65	7.19	1 [Reference]	/	1 [Reference]	/	1 [Reference]	/
Yes	101	5.13	0.68 (0.46,1.01)	0.056	0.64 (0.43,0.95)	0.027	0.59 (0.39,0.90)	0.013
**Non-cancer mortality**								
No	82	9.48	1 [Reference]	/	1 [Reference]	/	1 [Reference]	/
Yes	225	10.55	1.06 (0.72,1.57)	0.766	0.94 (0.67,1.32)	0.717	1.01 (0.70,1.46)	0.939
**Frailty index > 0.2 (Frail)**								
**All-cause mortality**								
No	251	37.57	1 [Reference]	/	1 [Reference]	/	1 [Reference]	/
Yes	458	35.25	1.03 (0.79,1.35)	0.836	0.90 (0.68,1.21)	0.494	0.95 (0.73,1.23)	0.682
**Cancer mortality**								
No	99	14.44	1 [Reference]	/	1 [Reference]	/	1 [Reference]	/
Yes	140	9.57	0.71 (0.48,1.04)	0.082	0.69 (0.45,1.03)	0.072	0.76 (0.51,1.13)	0.170
**Non-cancer mortality**								
No	152	23.12	1 [Reference]	/	1 [Reference]	/	1 [Reference]	/
Yes	318	25.69	1.23 (0.91,1.67)	0.179	1.04 (0.74,1.46)	0.834	1.07 (0.78,1.46)	0.684

Model 1 was adjusted for age, gender, race/ethnicity, education level, and marital status; Model 2 was additionally adjusted for smoking, alcohol consumption, HEI-2015, physical activity, hypertension, hyperlipidemia and diabetes history, CCI, and BMI or frailty index (if not already stratified). CVD, cardiovascular disease; BMI, body mass index; HR, hazard ratio; CI, confidence interval; HEI, Healthy Eating Index; CCI, Charlson Comorbidity Index.

## 4 Discussion

This cohort study discovered that a high BMI is linked to an increased risk of frailty in 3,932 US cancer survivors. BMI < 25 kg/m^2^ and FI > 0.2 were associated with an increased risk of death. Additionally, the use of dietary supplement can reduce the risk of death in cancer survivors with BMI < 25 kg/m^2^ and/or FI ≤ 0.2.

BMI has long been a crucial indicator for assessing the nutritional status and prognosis of cancer patients (2, 10, 27–31). In the US, approximately 7.8% of incident cancers (123,300/1,570,975 cases) and 6.5% of cancer-related deaths (38,230/587,521 deaths) were caused by effects of excess body weight, alcohol consumption, physical inactivity, and unhealthy diet (32). Excess weight has become one of the leading preventable causes of cancers similar to tobacco use (30). However, recent studies have indicated that overweight or obese cancer patients often have better outcomes, a phenomenon known as the “obesity paradox.” A clinical study involving 250 cancer patients undergoing αPD-(L)1 checkpoint blockade found that obese patients had significantly improved progression-free survival (PFS) (median: 237 versus 141 days, *p* = 0.0034) and overall survival (OS) (median: 523 versus 361 days, *p* = 0.0492) compared to non-obese patients (33). Similar survival advantages for obese patients were also found in studies by Naik GS (34) and Cortellini A (35). Obese patients have more energy reserves when facing cancer treatment, while malnutrition, underweight, and cachexia may impair immune function and surveillance, facilitating infections, treatment-related toxicity, recurrence, and distant metastasis (36). In this study, for the group with BMI ≥ 25 kg/m^2^, despite the presence of some individuals who may be frail due to obesity or resistant to treatment, greater nutritional reserves and tolerance to therapy might be one of the reasons for the overall better prognosis compared to the group with BMI < 25 kg/m^2^. This is consistent with the “obesity paradox.”

Considering the limited reliability of BMI in predicting clinical outcomes, frailty emerges as a more comprehensive measure linking patient health status with prognosis. Frailty is described as a complex, multidimensional, and recurring state of decreased physiologic reserve that leads to reduced resilience and adaptability, and heightened susceptibility to stressors (17). The numerous indicators used for calculating the FI are closely associated with the risk of mortality. Research indicates that cognitive decline can affect patients’ treatment decisions and adherence, thereby impacting the management and prognosis of diseases (37). Difficulties in activities of daily living reflect a decline in physical function, which may lead to a reduced quality of life and limited treatment options (38). The presence of depressive symptoms, such as low mood, fatigue, or loss of appetite, not only directly affects the patient’s mental health but also results in decreased treatment compliance (39). Comorbid conditions, such as heart disease, diabetes, arthritis, etc., may interact with cancer and its treatment, potentially increasing the risk of mortality (25). Changes in physical performance and anthropometric measurements may be signs of declining bodily functions and malnutrition (40). Laboratory values can reflect systemic health issues that can directly affect the survival rates of cancer patients (41). Finally, higher rates of hospitalization and healthcare utilization often indicate more severe health issues, which can lead to higher mortality rates (42). As a result, cancer patients with a high FI face a higher risk of mortality due to cumulative deficits in these multiple areas.

This study has revealed a correlation between higher BMI values and an increased risk of frailty in cancer patients. The correlation remains significant even after accounting for various health and socioeconomic factors. Obesity is associated with decreased muscle mass and strength, which can adversely affect overall bodily functions and resilience against external stressors (43). Obesity’s link with mental health issues, particularly depression, is well-documented and may further compound the effects of frailty (44, 45). Additionally, the limited physical activity and heightened risk of chronic diseases associated with obesity also contribute to reduced quality of life and self-care capabilities (46).

Cancer is commonly regarded as a catabolic disease. Tumors can modify a patient’s metabolism, resulting in increased energy and protein consumption, which can lead to malnutrition. Chemotherapy, radiotherapy, and other treatments can cause adverse effects such as appetite loss, taste changes, nausea, and vomiting, which can further exacerbate malnutrition. Given these circumstances, many cancer patients choose to take additional dietary supplements (such as vitamins, minerals, amino acids, herbs, and other similar components) (47–49). Studies suggest that appropriate nutritional interventions can improve the overall nutritional status of cancer patients, reduce complications, and potentially enhance quality of life (50–54). A randomized clinical trial involved 100 colorectal cancer patients undergoing adjuvant chemotherapy after curative surgery, where they were randomly assigned to receive either probiotics or a placebo postoperatively. The results showed that probiotics significantly reduced gastrointestinal reactions and helped balance intestinal flora (51). A study involving 128 gastrointestinal cancer patients receiving chemotherapy confirmed that fish oil-enriched nutrition could increase skeletal muscle and lean body mass, prevent the rise in serum CRP levels, and thereby improve chemotherapy tolerance (52). Additionally, a prospective cohort study of 247 survivors of colorectal cancer identified longitudinal associations between the consumption of macronutrients and micronutrients and the metabolic products of the tryptophan-kynurenine pathway. These associations may have potential implications for improving the health-related quality of life (HRQoL) of survivors (53). Another cohort study conducted on 30,239 individuals from the UK Biobank showed that cancer patients who regularly used dietary supplement (including vitamins, minerals, or non-vitamin non-mineral supplement) after diagnosis had a slightly lower risk of all-cause and cancer-specific mortality. This effect was particularly significant for non-vitamin non-mineral supplement, which showed a significant reduction in the risk of all-cause mortality (54).

However, research indicates that nearly 30% of patients do not inform their healthcare providers about their use of dietary supplements or other alternative treatments (55), leading to ambiguity in the clinical management and scientific research of dietary supplement application. This research, based on data from standardized questionnaires in the NHANES database, confirmed that the addition of dietary supplement is beneficial for non-obese and/or non-frail patient prognosis. However, in the overweight, obese, or frail population, dietary supplement cannot improve prognosis. The study further confirms that the impact of dietary supplements on cancer survivors is affected by BMI and FI through PSM and sensitivity analyses. This suggests that the efficacy of dietary supplement is not constant but is influenced by individual health conditions and other factors. A meta-analysis investigating the efficacy of probiotics, prebiotics, and synbiotics in treating anxiety revealed that the intervention group exhibited a significant reduction in anxiety scores compared to the placebo group within subgroups characterized by mental issues. However, no significant difference in anxiety scores was found between the two groups within subgroups characterized by physical problems or perfectly healthy. Similar disparities in treatment outcomes were also identified in subgroup analyses that utilized gastrointestinal symptoms or region as grouping variables (56). Another meta-analysis, based on individual participant data, synthesized the impact of small-quantity lipid-based nutrient supplements (SQ-LNSs) on child growth. The results revealed that the SQ-LNSs intervention had a more pronounced effect on child development within subgroups characterized by a greater prevalence of stunting, lower socioeconomic status, higher incidence of acute malnutrition, or elevated rates of anemia (57). These findings advocate for tailored recommendations regarding dietary supplement usage based on individual characteristics.

However, this study has several limitations. The analyses did not consider the type, dosage, and duration of dietary supplement use, which could have an impact on the results. Additionally, the study did not address more clinical details such as the types of cancer, anti-tumor medications, comorbid medications, and their duration, due to the limited types of data available in the NHANES database. Finally, it is important to note that the 49 items used to calculate the FI in this study are just one of many. This highlights the need for the development of more FI survey forms that are tailored to different patient groups and clinical scenarios. Before recommending dietary supplement to cancer survivors, it is important to take into account the overall health status, nutritional needs, and potential risks and benefits. For frail cancer patients, it is crucial to develop personalized treatment plans. This should include adjusting medication dosages, providing appropriate nutritional support, and implementing comprehensive rehabilitation programs.

## 5 Conclusion

Utilizing data from the NHANES and the NCHS, along with the 49-items, this study observed that among cancer survivors, a positive correlation was noted between BMI and FI. BMI < 25 kg/m^2^ and FI > 0.2 were associated with heightened risks of all-cause mortality and cancer-related mortality. The administration of dietary supplements appears to confer benefits to patients with a BMI < 25 kg/m2 and FI ≤ 0.2. However, more investigations are warranted to determine the optimal type, dosage form, and duration of usage of dietary supplement, as well as the characteristics of the intended population in the future.

## Data availability statement

Publicly available datasets were analyzed in this study. This data can be found here: National Health and Nutrition Examination Survey https://www.cdc.gov/nchs/nhanes/index.htm.

## Ethics statement

The studies involving humans were approved by the National Center for Health Statistics Ethics Review Board. The studies were conducted in accordance with the local legislation and institutional requirements. The participants provided their written informed consent to participate in this study.

## Author contributions

MZ: Methodology, Software, Writing – original draft. JW: Methodology, Software, Writing – original draft. XL: Visualization, Writing – original draft. LZ: Resources, Writing – original draft. YZ: Formal analysis, Writing – original draft. ZW: Data curation, Writing – original draft. JZ: Validation, Writing – original draft. YF: Conceptualization, Writing – original draft. ZQ: Project administration, Supervision, Writing – review & editing.
