# Supplementary material for: Association between dietary supplement use and mortality in cancer survivors with different body mass index and frailty status: a cohort study
Source: Front Nutr. 2024 May 1;11:1395362. doi: 10.3389/fnut.2024.1395362 (PMC11094810; doi:10.3389/fnut.2024.1395362)
Supplement: Supplementary file 1 [file Data_Sheet_1.pdf]

Supplementary Material

1 Supplementary Figures

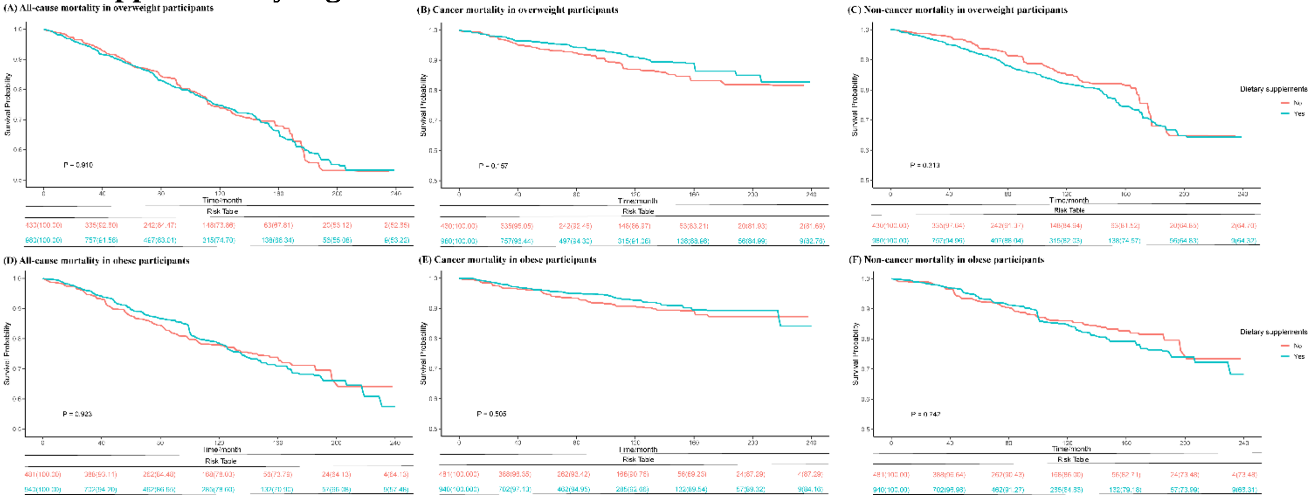

Supplementary Figure 1. Kaplan-Meier survival curves between dietary supplements and all-cause, cancer, and non-cancer mortality in 25 ≤ BMI < 30 km/m^2 and BMI ≥ 30 km/m^2 group. Abbreviation: BMI, body mass index.

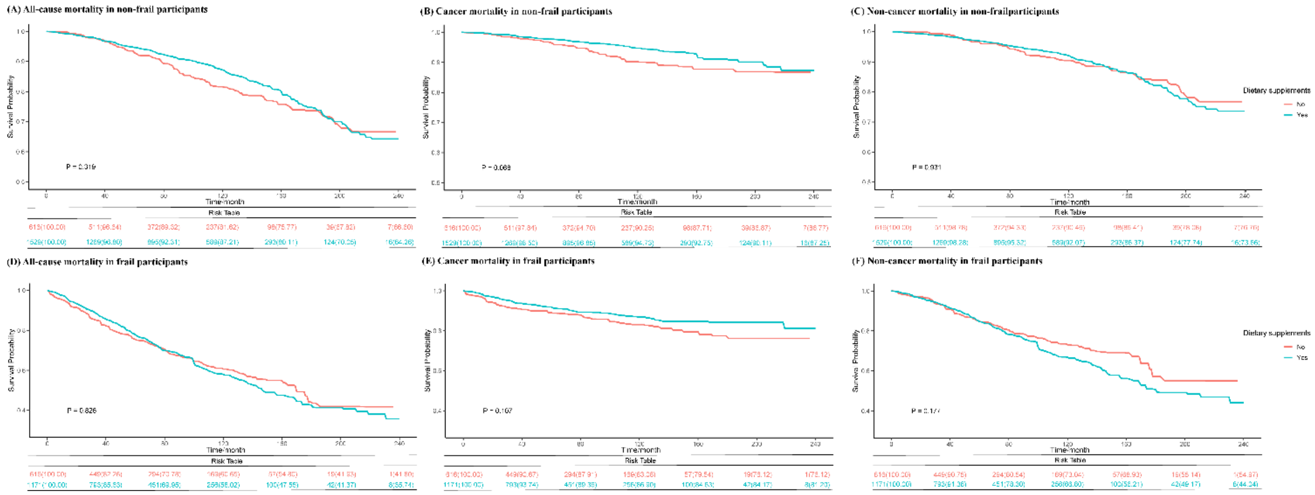

Supplementary Figure 2. Kaplan-Meier survival curves between dietary supplements and all-cause, cancer, and non-cancer mortality in non-frail and frail group.

2 Supplementary Tables

Supplementary Table 1. Variables in the 49-Item Frailty Index and Their Respective Scorings.

Variable	Scoring
Cognition	
1. Experience confusion/memory problems	Yes = 1, No = 0
Dependence	
2. Managing money	Difficulty = 1, No Difficulty = 0
3. Stooping, crouching, kneeling	Difficulty = 1, No Difficulty = 0
4. Lifting or carrying	Difficulty = 1, No Difficulty = 0
5. House chore	Difficulty = 1, No Difficulty = 0
6. Preparing meals	Difficulty = 1, No Difficulty = 0
7. Standing up from armless chair	Difficulty = 1, No Difficulty = 0
8. Getting in and out of bed difficulty	Difficulty = 1, No Difficulty = 0
9. Using fork, knife, drinking from cup	Difficulty = 1, No Difficulty = 0
10. Dressing yourself	Difficulty = 1, No Difficulty = 0
11. Standing for long periods difficulty	Difficulty = 1, No Difficulty = 0
12. Grasp/holding small objects	Difficulty = 1, No Difficulty = 0
13. Attending social event	Difficulty = 1, No Difficulty = 0
14. Push or pull large objects	Difficulty = 1, No Difficulty = 0
15. Walking for a quarter mile difficulty	Difficulty = 1, No Difficulty = 0
16. Walking up 10 steps difficulty	Difficulty = 1, No Difficulty = 0
Depressive Symptoms	
17. Have little interest in doing things	Nearly every day = 1, More than half the days = 0.66, Several days = 0.33, Not at all = 0
18. Feeling down, depressed, or hopeless	Nearly every day = 1, More than half the days = 0.66, Several days = 0.33, Not at all = 0
19. Trouble sleeping or sleeping too much	Nearly every day = 1, More than half the days = 0.66, Several days = 0.33, Not at all = 0
20. Feeling tired or having little energy	Nearly every day = 1, More than half the days = 0.66, Several days = 0.33, Not at all = 0

Variable	Scoring
21. Poor appetite or overeating	Nearly every day = 1, More than half the days = 0.66, Several days = 0.33, Not at all = 0
22. Feeling bad about yourself	Nearly every day = 1, More than half the days = 0.66, Several days = 0.33, Not at all = 0
23. Trouble concentrating on things	Nearly every day = 1, More than half the days = 0.66, Several days = 0.33, Not at all = 0
Comorbidities	
24. Arthritis	Yes = 1, Suspect = 0.5 No = 0
25. Thyroid problems	Yes = 1, Suspect = 0.5 No = 0
26. Chronic bronchitis	Yes = 1, Suspect = 0.5 No = 0
27. Cancer	Yes = 1, Suspect = 0.5 No = 0
28. Congestive heart failure	Yes = 1, Suspect = 0.5 No = 0
29. Coronary heart disease	Yes = 1, Suspect = 0.5 No = 0
30. Angina	Yes = 1, Suspect = 0.5 No = 0
31. Heart attack	Yes = 1, Suspect = 0.5 No = 0
32. Stroke	Yes = 1, Suspect = 0.5 No = 0
33. Blood pressure	Yes = 1, Suspect = 0.5 No = 0
34. Diabetes	Yes = 1, Suspect = 0.5 No = 0
35. weak/failing kidneys	Yes = 1, Suspect = 0.5 No = 0
36. Urinary Leakage	Yes = 1, Suspect = 0.5 No = 0
Hospital Utilization and Access to Care	
37. Self-rated health	Fair, poor = 1, Excellent, Very good, good = 0
38. Health now compared with 1 year ago	Worse = 1, About the same, better = 0
39. Overnight hospital patient in past year	Yes = 1, No = 0
40. Frequency of health care use during past year	None = 0, 1–5 = 0,5, More than 5 = 1
41. Number of prescribed medications	None = 0, 1–4 = 0.5, 5 and more = 1
Physical Performance and	

Variable	Scoring	
Anthropometry		
42. Body mass index	<18.5 km/m^2 or ≥30 km/m^2 = 1<br>>25 km/m^2 and <30 km/m^2 = 0.5<br>18.5–25 km/m^2 = 0	
43. Handgrip strength	MALE:<br>For BMI ≤ 24 km/m^2,<br>GS ≤ 29<br>For BMI 24.1–28 km/m^2,<br>GS ≤ 30<br>For BMI >28 km/m^2,<br>GS ≤ 32 = 1	FEMALE:<br>For BMI ≤ 23 km/m^2, GS ≤ 17<br>For BMI 23.1–26 km/m^2,<br>GS ≤ 17.3<br>For BMI 26.1–29 km/m^2,<br>GS ≤ 18<br>For BMI>29 km/m^2,<br>GS ≤ 21 = 1
Laboratory Values		
44. Glycohemoglobin (%)	0%–5.7% = 0, >5.7% = 1	
45. Red blood cell count (million cells/μL)	M: 4.7–6.1 = 0, Other = 1	F: 4.2–5.4 = 0, Other = 1
46. Hemoglobin (g/dL)	M: 13.5–18 = 0, Other = 1	F: 12–16 = 0, Other = 1
47. Red cell distribution width (%)	11.6–14.6 = 0, Other = 1	
48. Lymphocyte percent (%)	20–40 = 0, Other = 1	
49. Segmented neutrophils percent (%)	40–80 = 0, Other = 1	

Supplementary Table 2. Collinearity analysis.

Variables	VIF
Age	3.55
Gender	1.86
Race/ethnicity	6.52
Education	3.10
Marital status	2.41
Smoking	2.58
Alcohol consumption	1.82
BMI	2.66
Frailty index	2.24
HEI–2015	2.22
Physical activity	1.61
Hypertension	2.10
Hyperlipidemia	1.63
Diabetes	1.65
CCI	1.75
Dietary supplements	1.53

Abbreviations: VIF, variance inflation factor; BMI, body mass index; HEI, Healthy Eating Index; CCI, Charlson Comorbidity Index.

Supplementary Table 3. Survey-weighted associations between dietary supplements and all-cause, cancer and non-cancer mortality among cancer survivors in different BMI (kg/m^2) groups.

	Death, n	Weighted death (%)	Univariable model		Model 1		Model 2	
			HR (*95%CI*)	*P* value	*HR* (*95%CI*)	*P* value	*HR* (*95%CI*)	*P* value
**BMI < 25 kg/m^2**								
**All-cause mortality**								
No	133	32.58	1[Reference]	/	1[Reference]	/	1[Reference]	/
Yes	274	24.87	0.69(0.51,0.94)	0.017	0.60(0.45,0.81)	< 0.001	0.63(0.47,0.84)	0.002
**Cancer mortality**								
No	54	13.65	1[Reference]	/	1[Reference]	/	1[Reference]	/
Yes	79	6.77	0.47(0.28,0.77)	0.003	0.47(0.28,0.77)	0.003	0.48(0.29,0.80)	0.005
**Non-cancer mortality**								
No	79	18.93	1[Reference]	/	1[Reference]	/	1[Reference]	/
Yes	195	18.10	0.85(0.57,1.28)	0.438	0.69(0.46,1.05)	0.083	0.75(0.50,1.13)	0.167
**25 ⩽ BMI < 30 kg/m^2**								
**All-cause mortality**								
No	145	25.76	1[Reference]	/	1[Reference]	/	1[Reference]	/
Yes	313	25.77	1.01(0.79,1.30)	0.911	0.95(0.75,1.21)	0.704	1.13(0.87,1.45)	0.363
**Cancer mortality**								
No	62	10.91	1[Reference]	/	1[Reference]	/	1[Reference]	/
Yes	91	8.11	0.76(0.53,1.10)	0.151	0.75(0.51,1.11)	0.748	0.85(0.57,1.27)	0.433
**Non-cancer mortality**								
No	83	14.85	1[Reference]	/	1[Reference]	/	1[Reference]	/
Yes	222	17.67	1.20(0.84,1.71)	0.317	1.10(0.78,1.56)	0.582	1.30(0.89,1.89)	0.168
**BMI ⩾ 30 kg/m^2**								
**All-cause mortality**								
No	130	20.75	1[Reference]	/	1[Reference]	/	1[Reference]	/
Yes	216	19.15	0.98(0.70,1.38)	0.925	0.90(0.62,1.29)	0.552	0.91(0.64,1.28)	0.581
**Cancer mortality**								
No	52	7.87	1[Reference]	/	1[Reference]	/	1[Reference]	/
Yes	78	6.27	0.85(0.52,1.37)	0.498	0.80(0.49,1.32)	0.382	0.89(0.56,1.42)	0.623
**Non-cancer mortality**								
No	78	12.88	1[Reference]	/	1[Reference]	/	1[Reference]	/
Yes	138	12.88	1.07(0.73,1.57)	0.741	0.95(0.61,1.50)	0.838	0.90(0.57,1.42)	0.658

Model 1 was adjusted for age, gender, race/ethnicity, education level, and marital status;
Model 2 was additionally adjusted for smoking, alcohol consumption, HEI-2015, physical activity, frailty index, hypertension, hyperlipidemia and diabetes history, and CCI.
Abbreviations: CVD, cardiovascular disease; BMI, body mass index; *HR*, hazard ratio; *CI*, confidence interval; HEI, Healthy Eating Index; CCI, Charlson Comorbidity Index.

Supplementary Table 4. Survey-weighted associations between dietary supplements and all-cause, cancer and non-cancer mortality among cancer survivors in different frailty groups.

	Death, n	Weighted death (%)	Univariable model		Model 1		Model 2	
			HR (*95%CI*)	*P* value	*HR* (*95%CI*)	*P* value	*HR* (*95%CI*)	*P* value
**Frailty index ≤ 0.2 (Non-frail)**								
**All-cause mortality**								
No	151	17.70	1[Reference]	/	1[Reference]	/	1[Reference]	/
Yes	332	16.08	0.87(0.66,1.15)	0.321	0.78(0.60,1.00)	0.051	0.77(0.60,0.99)	0.041
**Cancer mortality**								
No	66	7.82	1[Reference]	/	1[Reference]	/	1[Reference]	/
Yes	102	5.49	0.68(0.46,1.01)	0.057	0.63(0.43,0.95)	0.025	0.59(0.39,0.89)	0.011
**Non-cancer mortality**								
No	85	9.88	1[Reference]	/	1[Reference]	/	1[Reference]	/
Yes	230	10.59	1.02(0.69,1.50)	0.931	0.89(0.63,1.26)	0.524	0.95(0.65,1.38)	0.782
**Frailty index > 0.2 (Frail)**								
**All-cause mortality**								
No	257	25.86	1[Reference]	/	1[Reference]	/	1[Reference]	/
Yes	471	23.23	1.03(0.79,1.34)	0.826	0.90(0.69,1.19)	0.462	0.95(0.73,1.22)	0.672
**Cancer mortality**								
No	102	10.57	1[Reference]	/	1[Reference]	/	1[Reference]	/
Yes	146	7.08	0.72(0.49,1.06)	0.096	0.69(0.46,1.04)	0.073	0.76(0.51,1.13)	0.175
**Non-cancer mortality**								
No	155	15.29	1[Reference]	/	1[Reference]	/	1[Reference]	/
Yes	325	16.15	1.23(0.91,1.66)	0.179	1.03(0.74,1.43)	0.854	1.07(0.79,1.44)	0.683

Model 1 was adjusted for age, gender, race/ethnicity, education level, and marital status;
Model 2 was additionally adjusted for smoking, alcohol consumption, HEI-2015, physical activity, BMI, hypertension, hyperlipidemia and diabetes history, and CCI.
Abbreviations: CVD, cardiovascular disease; BMI, body mass index; *HR*, hazard ratio; *CI*, confidence interval; HEI, Healthy Eating Index; CCI, Charlson Comorbidity Index.

Supplementary Table 5. Sensitivity analysis of the associations between dietary supplements and all-cause, cancer, and non-cancer mortality among cancer survivors in different BMI (kg/m^2) or frailty groups after Propensity Score Matching.

	Death, n	Weighted death (%)	Univariable model		Model 1		Model 2	
			HR (*95%CI*)	*P* value	*HR* (*95%CI*)	*P* value	*HR* (*95%CI*)	*P* value
**BMI < 25** km/m^2								
**All-cause mortality**								
No	138	32.58	1[Reference]	/	1[Reference]	/	1[Reference]	/
Yes	143	23.19	0.71(0.49,1.02)	0.061	0.46(0.33,0.62)	< 0.001	0.55(0.41,0.76)	< 0.001
**Cancer mortality**								
No	54	13.65	1[Reference]	/	1[Reference]	/	1[Reference]	/
Yes	35	5.57	0.41(0.23,0.73)	0.002	0.37(0.21,0.67)	< 0.001	0.39(0.19,0.78)	0.008
**Non-cancer mortality**								
No	79	18.93	1[Reference]	/	1[Reference]	/	1[Reference]	/
Yes	108	17.62	0.92(0.58,1.47)	0.725	0.50(0.33,0.76)	0.001	0.68(0.46,0.99)	0.042
**25 ⩽ BMI < 30** km/m^2								
**All-cause mortality**								
No	145	25.76	1[Reference]	/	1[Reference]	/	1[Reference]	/
Yes	162	26.55	1.16(0.90,1.49)	0.249	0.94(0.70,1.28)	0.712	1.33(0.96,1.84)	0.086
**Cancer mortality**								
No	62	10.91	1[Reference]	/	1[Reference]	/	1[Reference]	/
Yes	40	8.53	0.86(0.55,1.33)	0.487	0.77(0.42,1.43)	0.408	1.05(0.53,2.09)	0.879
**Non-cancer mortality**								
No	83	14.85	1[Reference]	/	1[Reference]	/	1[Reference]	/
Yes	122	18.03	1.39(0.99,1.94)	0.059	1.08(0.73,1.60)	0.696	1.48(0.94,2.33)	0.090
**BMI ⩾ 30** km/m^2								
**All-cause mortality**								
No	130	20.75	1[Reference]	/	1[Reference]	/	1[Reference]	/
Yes	73	15.84	0.88(0.59,1.31)	0.520	0.54(0.33,0.86)	0.010	0.58(0.36,0.93)	0.023
**Cancer mortality**								
No	52	7.87	1[Reference]	/	1[Reference]	/	1[Reference]	/
Yes	27	5.76	0.82(0.46,1.48)	0.515	0.52(0.25,1.07)	0.075	0.68(0.30,1.56)	0.361
**Non-cancer mortality**								
No	78	12.88	1[Reference]	/	1[Reference]	/	1[Reference]	/
Yes	46	10.09	0.91(0.56,1.47)	0.701	0.55(0.32,0.94)	0.028	0.54(0.33,0.89)	0.015
**Frailty index ⩽ 0.2 (Non-frail)**								
**All-cause mortality**								
No	151	17.70	1[Reference]	/	1[Reference]	/	1[Reference]	/
Yes	191	16.27	1.00(0.76,1.33)	0.976	0.76(0.57,1.02)	0.071	0.76(0.57,1.02)	0.818
**Cancer mortality**								
No	66	7.82	1[Reference]	/	1[Reference]	/	1[Reference]	/
Yes	58	6.13	0.85(0.55,1.31)	0.459	0.76(0.46,1.26)	0.285	0.87(0.48,1.56)	0.239
**Non-cancer mortality**								
No	85	9.88	1[Reference]	/	1[Reference]	/	1[Reference]	/

Yes	133	10.14	1.13(0.76,1.66)	0.548	0.79(0.55,1.11)	0.175	1.11(0.74,1.67)	0.609
**Frailty index > 0.2 (Frail)**								
**All-cause mortality**								
No	257	37.99	1[Reference]	/	1[Reference]	/	1[Reference]	/
Yes	187	36.23	1.13(0.84,1.52)	0.424	0.71(0.51,0.98)	0.037	0.78(0.56,1.09)	0.151
**Cancer mortality**								
No	102	14.65	1[Reference]	/	1[Reference]	/	1[Reference]	/
Yes	44	8.19	0.62(0.39,0.99)	0.047	0.47(0.27,0.83)	0.009	0.57(0.29,1.09)	0.090
**Non-cancer mortality**								
No	155	23.34	1[Reference]	/	1[Reference]	/	1[Reference]	/
Yes	143	28.05	1.47(1.06,2.02)	0.020	0.82(0.60,1.12)	0.211	0.88(0.65,1.19)	0.392

Model 1 was adjusted for age, gender, race/ethnicity, education level, and marital status;
Model 2 was additionally adjusted for smoking, alcohol consumption, HEI-2015, physical activity, frailty index, hypertension, hyperlipidemia and diabetes history, and CCI.
Abbreviations: CVD, cardiovascular disease; BMI, body mass index; *HR*, hazard ratio; *CI*, confidence interval; HEI, Healthy Eating Index; CCI, Charlson Comorbidity Index.

Supplementary Table 6. Sensitivity analysis of the associations between dietary supplements and all-cause, cancer, and non-cancer mortality among cancer survivors in different BMI (kg/m^2) or frailty groups after exclusion deaths with a follow-up period of fewer than two years.

	Death, n	Weighted death (%)	Univariable model		Model 1		Model 2	
			HR (*95%CI*)	*P* value	*HR* (*95%CI*)	*P* value	*HR* (*95%CI*)	*P* value
**BMI < 25** km/m^2								
**All-cause mortality**								
No	97	28.38	1[Reference]	/	1[Reference]	/	1[Reference]	/
Yes	224	22.92	0.76(0.54,1.07)	0.111	0.62(0.45,0.86)	0.005	0.63(0.45,0.87)	0.005
**Cancer mortality**								
No	31	9.10	1[Reference]	/	1[Reference]	/	1[Reference]	/
Yes	55	5.51	0.59(0.34,1.03)	0.065	0.53(0.30,0.94)	0.031	0.46(0.24,0.86)	0.015
**Non-cancer mortality**								
No	66	19.28	1[Reference]	/	1[Reference]	/	1[Reference]	/
Yes	169	17.41	0.84(0.54,1.30)	0.430	0.67(0.43,1.04)	0.071	0.72(0.46,1.12)	0.146
**25 ⩽ BMI < 30** km/m^2								
**All-cause mortality**								
No	120	24.49	1[Reference]	/	1[Reference]	/	1[Reference]	/
Yes	264	24.08	0.98(0.73,1.31)	0.886	0.91(0.69,1.19)	0.485	1.08(0.81,1.45)	0.592
**Cancer mortality**								
No	49	9.56	1[Reference]	/	1[Reference]	/	1[Reference]	/
Yes	71	6.91	0.73(0.45,1.19)	0.207	0.68(0.42,1.12)	0.133	0.78(0.46,1.32)	0.351
**Non-cancer mortality**								
No	71	14.93	1[Reference]	/	1[Reference]	/	1[Reference]	/
Yes	193	17.17	1.14(0.78,1.66)	0.502	1.06(0.74,1.51)	0.766	1.25(0.85,1.85)	0.255
**BMI ⩾ 30** km/m^2								
**All-cause mortality**								
No	112	18.67	1[Reference]	/	1[Reference]	/	1[Reference]	/
Yes	180	18.38	1.01(0.72,1.44)	0.935	0.93(0.62,1.37)	0.703	0.92(0.62,1.36)	0.685
**Cancer mortality**								
No	39	5.97	1[Reference]	/	1[Reference]	/	1[Reference]	/
Yes	61	5.53	0.85(0.52,1.37)	0.498	0.80(0.49,1.32)	0.382	0.89(0.56,1.42)	0.623
**Non-cancer mortality**								
No	73	12.70	1[Reference]	/	1[Reference]	/	1[Reference]	/
Yes	119	12.82	1.04(0.69,1.57)	0.851	0.92(0.56,1.52)	0.757	0.85(0.52,1.40)	0.528
**Frailty index ⩽ 0.2 (Non-frail)**								
**All-cause mortality**								
No	141	18.02	1[Reference]	/	1[Reference]	/	1[Reference]	/
Yes	305	15.82	0.84(0.62,1.12)	0.236	0.74(0.56,0.97)	0.028	0.74(0.56,0.97)	0.028
**Cancer mortality**								
No	58	7.53	1[Reference]	/	1[Reference]	/	1[Reference]	/
Yes	92	5.19	0.68(0.46,1.01)	0.057	0.63(0.43,0.95)	0.025	0.59(0.39,0.89)	0.011
**Non-cancer mortality**								
No	83	10.50	1[Reference]	/	1[Reference]	/	1[Reference]	/

Yes	213	10.63	0.96(0.64,1.43)	0.829	0.84(0.59,1.20)	0.337	0.91(0.61,1.34)	0.627
**Frailty index > 0.2 (Frail)**								
**All-cause mortality**								
No	188	31.73	1[Reference]	/	1[Reference]	/	1[Reference]	/
Yes	363	33.20	1.14(0.85,1.53)	0.369	1.00(0.74,1.35)	0.989	1.03(0.78,1.37)	0.838
**Cancer mortality**								
No	61	8.92	1[Reference]	/	1[Reference]	/	1[Reference]	/
Yes	95	7.61	0.92(0.58,1.46)	0.729	0.90(0.57,1.42)	0.663	1.01(0.64,1.61)	0.953
**Non-cancer mortality**								
No	127	22.81	1[Reference]	/	1[Reference]	/	1[Reference]	/
Yes	268	25.60	1.23(0.88,1.72)	0.222	1.04(0.72,1.50)	0.837	1.05(0.75,1.47)	0.793

Model 1 was adjusted for age, gender, race/ethnicity, education level, and marital status;
Model 2 was additionally adjusted for smoking, alcohol consumption, HEI-2015, physical activity, frailty index, hypertension, hyperlipidemia and diabetes history, and CCI.
Abbreviations: CVD, cardiovascular disease; BMI, body mass index; *HR*, hazard ratio; *CI*, confidence interval; HEI, Healthy Eating Index; CCI, Charlson Comorbidity Index.

Supplementary Table 7. Sensitivity analysis of the associations between dietary supplements and all-cause, cancer, and non-cancer mortality among cancer survivors in different BMI (kg/m^2) or frailty groups after the exclusion participants over 80 years of age.

	Death, n	Weighted death (%)	Univariable model		Model 1		Model 2	
			HR (*95%CI*)	*P* value	*HR* (*95%CI*)	*P* value	*HR* (*95%CI*)	*P* value
**BMI < 25** km/m^2								
**All-cause mortality**								
No	117	30.80	1[Reference]	/	1[Reference]	/	1[Reference]	/
Yes	217	22.26	0.65(0.47,0.91)	0.010	0.60(0.43,0.83)	0.002	0.65(0.47,0.89)	0.008
**Cancer mortality**								
No	47	12.63	1[Reference]	/	1[Reference]	/	1[Reference]	/
Yes	66	6.15	0.46(0.27,0.80)	0.005	0.49(0.28,0.86)	0.013	0.53(0.30,0.95)	0.033
**Non-cancer mortality**								
No	70	18.17	1[Reference]	/	1[Reference]	/	1[Reference]	/
Yes	151	16.11	0.79(0.51,1.21)	0.276	0.66(0.42,1.04)	0.073	0.74(0.48,1.15)	0.179
**25 ⩽ BMI < 30** km/m^2								
**All-cause mortality**								
No	129	24.12	1[Reference]	/	1[Reference]	/	1[Reference]	/
Yes	262	23.55	0.98(0.76,1.27)	0.896	0.90(0.71,1.15)	0.397	1.09(0.84,1.42)	0.500
**Cancer mortality**								
No	58	10.70	1[Reference]	/	1[Reference]	/	1[Reference]	/
Yes	84	7.92	0.75(0.52,1.09)	0.136	0.73(0.49,1.08)	0.118	0.85(0.55,1.29)	0.435
**Non-cancer mortality**								
No	71	13.42	1[Reference]	/	1[Reference]	/	1[Reference]	/
Yes	178	15.63	1.16(0.79,1.72)	0.444	1.04(0.72,1.50)	0.826	1.28(0.87,1.90)	0.216
**BMI ⩾ 30** km/m^2								
**All-cause mortality**								
No	123	19.97	1[Reference]	/	1[Reference]	/	1[Reference]	/
Yes	199	18.19	0.97(0.69,1.38)	0.880	0.91(0.62,1.33)	0.623	0.91(0.63,1.31)	0.614
**Cancer mortality**								
No	50	7.51	1[Reference]	/	1[Reference]	/	1[Reference]	/
Yes	74	6.12	0.87(0.53,1.42)	0.573	0.85(0.51,1.43)	0.548	0.93(0.57,1.50)	0.760
**Non-cancer mortality**								
No	73	12.45	1[Reference]	/	1[Reference]	/	1[Reference]	/
Yes	125	12.08	1.04(0.70,1.55)	0.855	0.94(0.59,1.51)	0.805	0.88(0.55,1.42)	0.613
**Frailty index ⩽ 0.2 (Non-frail)**								
**All-cause mortality**								
No	134	16.65	1[Reference]	/	1[Reference]	/	1[Reference]	/
Yes	274	14.53	0.83(0.62,1.12)	0.229	0.75(0.58,0.97)	0.028	0.76(0.59,0.98)	0.035
**Cancer mortality**								
No	60	7.37	1[Reference]	/	1[Reference]	/	1[Reference]	/
Yes	91	5.19	0.69(0.46,1.03)	0.071	0.64(0.43,0.96)	0.031	0.61(0.41,0.92)	0.020
**Non-cancer mortality**								
No	74	9.28	1[Reference]	/	1[Reference]	/	1[Reference]	/

Yes	183	9.33	0.95(0.62,1.45)	0.813	0.83(0.57,1.20)	0.325	0.89(0.60,1.33)	0.572
**Frailty index > 0.2 (Frail)**								
**All-cause mortality**								
No	235	36.25	1[Reference]	/	1[Reference]	/	1[Reference]	/
Yes	404	33.52	0.61(0.41,0.92)	0.971	0.89(0.67,1.19)	0.436	0.94(0.72,1.23)	0.672
**Cancer mortality**								
No	95	14.11	1[Reference]	/	1[Reference]	/	1[Reference]	/
Yes	133	9.63	0.72(0.48,1.09)	0.118	0.72(0.47,1.10)	0.128	0.80(0.53,1.21)	0.290
**Non-cancer mortality**								
No	140	22.13	1[Reference]	/	1[Reference]	/	1[Reference]	/
Yes	271	23.89	1.19(0.86,1.63)	0.289	1.00(0.72,1.39)	0.998	1.04(0.76,1.42)	0.812

Model 1 was adjusted for age, gender, race/ethnicity, education level, and marital status;
Model 2 was additionally adjusted for smoking, alcohol consumption, HEI-2015, physical activity, frailty index, hypertension, hyperlipidemia and diabetes history, and CCI.
Abbreviations: CVD, cardiovascular disease; BMI, body mass index; *HR*, hazard ratio; *CI*, confidence interval; HEI, Healthy Eating Index; CCI, Charlson Comorbidity Index.

Supplementary Table 8. Sensitivity analysis of the associations between dietary supplements and all-cause, cancer, and non-cancer mortality among cancer survivors in different BMI (kg/m^2) or frailty groups without complex survey design.

	Death, n	Death, %	Univariable model		Model 1		Model 2	
			HR (*95%CI*)	*P* value	*HR* (*95%CI*)	*P* value	*HR* (*95%CI*)	*P* value
**BMI < 25** km/m^2								
**All-cause mortality**								
No	133	41.43	1[Reference]	/	1[Reference]	/	1[Reference]	/
Yes	274	35.13	0.79(0.64,0.97)	0.023	0.70(0.57,0.88)	0.002	0.70(0.56,0.87)	0.002
**Cancer mortality**								
No	54	16.82	1[Reference]	/	1[Reference]	/	1[Reference]	/
Yes	79	10.13	0.57(0.40,0.81)	0.001	0.59(0.41,0.84)	0.004	0.59(0.40,0.85)	0.005
**Non-cancer mortality**								
No	79	24.61	1[Reference]	/	1[Reference]	/	1[Reference]	/
Yes	195	25.00	0.93(0.72,1.21)	0.610	0.77(0.59,1.02)	0.066	0.79(0.60,1.05)	0.100
**25 ⩽ BMI < 30** km/m^2								
**All-cause mortality**								
No	145	33.72	1[Reference]	/	1[Reference]	/	1[Reference]	/
Yes	313	31.94	0.98(0.80,1.19)	0.819	0.85(0.69,1.04)	0.119	0.94(0.76,1.15)	0.533
**Cancer mortality**								
No	62	14.42	1[Reference]	/	1[Reference]	/	1[Reference]	/
Yes	91	9.29	0.67(0.48,0.92)	0.014	0.65(0.47,0.91)	0.011	0.71(0.51,1.00)	0.050
**Non-cancer mortality**								
No	83	19.30	1[Reference]	/	1[Reference]	/	1[Reference]	/
Yes	222	22.65	1.21(0.94,1.56)	0.141	0.99(0.77,1.29)	0.957	1.09(0.84,1.42)	0.511
**BMI ⩾ 30** km/m^2								
**All-cause mortality**								
No	130	27.03	1[Reference]	/	1[Reference]	/	1[Reference]	/
Yes	216	22.98	0.90(0.73,1.12)	0.355	0.80(0.64,1.01)	0.057	0.89(0.71,1.13)	0.353
**Cancer mortality**								
No	52	12.06	1[Reference]	/	1[Reference]	/	1[Reference]	/
Yes	78	8.30	0.81(0.57,1.16)	0.251	0.78(0.54,1.14)	0.199	0.85(0.59,1.25)	0.416
**Non-cancer mortality**								
No	78	16.22	1[Reference]	/	1[Reference]	/	1[Reference]	/
Yes	138	14.68	0.96(0.73,1.27)	0.782	0.81(0.61,1.09)	0.158	0.92(0.68,1.25)	0.599
**Frailty index ⩽ 0.2 (Non-frail)**								
**All-cause mortality**								
No	151	24.51	1[Reference]	/	1[Reference]	/	1[Reference]	/
Yes	332	21.71	0.86(0.71,1.04)	0.125	0.76(0.62,0.93)	0.007	0.76(0.62,0.93)	0.008
**Cancer mortality**								
No	66	10.71	1[Reference]	/	1[Reference]	/	1[Reference]	/
Yes	102	6.67	0.61(0.45,0.83)	0.002	0.57(0.41,0.79)	< 0.001	0.57(0.41,0.79)	< 0.001
**Non-cancer mortality**								
No	85	13.80	1[Reference]	/	1[Reference]	/	1[Reference]	/

Yes	230	15.04	1.05(0.82,1.35)	0.688	0.91(0.70,1.17)	0.453	0.93(0.72,1.20)	0.571
**Frailty index > 0.2 (Frail)**								
**All-cause mortality**								
No	257	41.72	1[Reference]	/	1[Reference]	/	1[Reference]	/
Yes	471	40.22	1.07(0.92,1.24)	0.410	0.88(0.75,1.03)	0.105	0.93(0.79,1.09)	0.361
**Cancer mortality**								
No	102	16.56	1[Reference]	/	1[Reference]	/	1[Reference]	/
Yes	146	12.47	0.82(0.63,1.05)	0.115	0.78(0.60,1.01)	0.058	0.82(0.62,1.06)	0.131
**Non-cancer mortality**								
No	155	25.16	1[Reference]	/	1[Reference]	/	1[Reference]	/
Yes	325	27.75	1.23(1.02,1.49)	0.032	0.93(0.76,1.13)	0.476	0.99(0.81,1.21)	0.913

Model 1 was adjusted for age, gender, race/ethnicity, education level, and marital status;

Model 2 was additionally adjusted for smoking, alcohol consumption, HEI-2015, physical activity, frailty index, hypertension, hyperlipidemia and diabetes history, and CCI.

Abbreviations: CVD, cardiovascular disease; BMI, body mass index; *HR*, hazard ratio; *CI*, confidence interval; HEI, Healthy Eating Index; CCI, Charlson Comorbidity Index.
